# Chemoradiotherapy versus surgery after neoadjuvant chemoimmunotherapy in patients with stage III NSCLC: a real-world multicenter retrospective study

**DOI:** 10.1007/s00262-024-03696-4

**Published:** 2024-05-07

**Authors:** Song Guan, Jifeng Sun, Yuan Wang, Sibei Han, Chen Chen, Dongsheng Yue, Yubei Huang, Kai Ren, Jun Wang, Jun Wang, Lujun Zhao

**Affiliations:** 1 Department of Radiation Oncology, Tianjin Medical University Cancer Institute & Hospital, National Clinical Research Center for Cancer, Tianjin’s Clinical Research Center for Cancer, Key Laboratory of Cancer Prevention and Therapy, Huan-Hu Xi Road, Ti-Yuan-Bei, He Xi District, Tianjin, 300060 China; 2 Department of Radiotherapy, Tianjin Cancer Hospital Airport Hospital, East 5Th Road, Tianjin Airport Economic District, Tianjin, 300308 China; 3 Department of Radiotherapy, The Fourth Hospital of Hebei Medical University, Hebei Clinical Research Center for Radiation Oncology, Shijiazhuang, 050011 China; 4 Department of Oncology, The 983Th Hospital of the PLA Joint Logistics Support Force, Tianjin, China; 5 Department of Lung Cancer, Tianjin Lung Cancer Center, National Clinical Research Center for Cancer, Key Laboratory of Cancer Prevention and Therapy, Tianjin’s Clinical Research Center for Cancer, Tianjin Medical University Cancer Institute and Hospital, Tianjin, China; 6 Department of Cancer Epidemiology and Biostatistics, Key Laboratory of Molecular Cancer Epidemiology (Tianjin), Tianjin Medical University Cancer Institute and Hospital, National Clinical Research Center for Cancer, Key Laboratory of Cancer Prevention and Therapy, Tianjin’s Clinical Research Center for Cancer, Tianjin, China

**Keywords:** Chemoradiotherapy, Neoadjuvant chemoimmunotherapy, Non-small cell lung cancer, Surgery

## Abstract

**Purpose:**

The optimal treatment after neoadjuvant chemoimmunotherapy for patients with stage III non-small cell lung cancer (NSCLC) is unclear. This study aimed at comparing the efficacy and safety of chemoradiotherapy and surgery after neoadjuvant chemoimmunotherapy in stage III NSCLC.

**Materials and methods:**

We conducted a real-world multicenter retrospective study on patients with stage III NSCLC who received surgery or chemoradiotherapy after neoadjuvant chemoimmunotherapy between October 2018 and December 2022. Progression-free survival (PFS) and overall survival (OS) were assessed from the initiation of neoadjuvant treatment and estimated by the Kaplan‒Meier method. Univariate and multivariate Cox regression models were used to examine potential prognostic factors. One-to-one propensity score matching (PSM) was used to further minimize confounding.

**Results:**

A total of 239 eligible patients were enrolled, with 104 (43.5%) receiving surgery and 135 (56.5%) receiving CRT. After 1:1 PSM, 1- and 2-year PFS rates in patients receiving radical surgery (rSurgery group) vs. patients receiving definitive cCRT (dCCRT group) were 80.0% vs. 79.2% and 67.2% vs. 53.1%, respectively (*P* = 0.774). One- and 2-year OS rates were 97.5% vs. 97.4% and 87.3% vs. 89.9%, respectively (*P* = 0.558). Patients in the dCCRT group had a numerically lower incidence of distant metastases compared to those in the rSurgery group (42.9% vs. 70.6%, *P* = 0.119). The incidence of treatment-related adverse events was similar in both groups, except that the incidence of grade 3/4 hematological toxicity was significantly higher in the dCCRT group (30.0% vs. 10.0%, *P* = 0.025).

**Conclusion:**

Following neoadjuvant chemoimmunotherapy, definitive concurrent chemoradiotherapy may achieve noninferior outcomes to radical surgery in stage III NSCLC.

**Supplementary Information:**

The online version contains supplementary material available at 10.1007/s00262-024-03696-4.

## Introduction

Stage III non-small cell lung cancer (NSCLC), which accounts for approximately one-third of NSCLC cases, represents a heterogeneous group for which optimal treatment is uncertain [1]. Based on the primary tumor extension and nodal involvement, stage III NSCLC is categorized into resectable, potentially resectable and unresectable disease [2]. Classically, the standard of care for patients with unresectable stage III NSCLC in the immunotherapy era is concurrent chemoradiotherapy (cCRT) followed by consolidation immunotherapy (the PACIFIC regimen) [3, 4]. However, the optimal sequence of immunotherapy and radiotherapy remains unclear, and upfront immunotherapy before chemoradiotherapy (CRT) is increasingly recommended to downsize tumor and increase the likelihood of success with subsequent definitive CRT [5, 6]. For patients with resectable stage III disease, the combination of immunotherapy and surgery could provide tremendous potential benefits and has led to the increasing application of neoadjuvant immunotherapy [7–9]. However, the impressive efficacy of neoadjuvant immunotherapy leads to questions regarding the conversion of unresectable status to resectable status. Several studies have shown the promising outcomes of neoadjuvant chemoimmunotherapy followed by surgery in patients with unresectable stage III NSCLC [10–12], suggesting that post-conversion surgical resection may play a role in this scenario. Interestingly, with a deeper understanding of immunotherapy, robust evidence has shown that the function and integrity of tumor-draining lymph nodes (dLNs) appear to correlate with the efficacy of immunotherapy [13–15]. Compared with radical surgery involving regional lymphadenectomy, chemoradiotherapy, while also impairing the dLNs, appears to preserve the function and integrity of dLNs better with the help of some predictive models, such as estimated radiation doses to immune cells (EDIC) [16, 17], which in turn may lead to a noninferior outcome to surgery. Considering that previous studies in the preimmunotherapy era failed to demonstrate superior PFS and OS in patients with stage IIIA (N2) and selected IIIB who received surgery after neoadjuvant chemotherapy compared to CRT [18, 19], we therefore conducted this real-world multicenter retrospective study to investigate the optimal treatment after neoadjuvant chemoimmunotherapy.

## Materials and methods

### Patient selection

Patients with stage III NSCLC who received surgery or CRT after neoadjuvant chemoimmunotherapy at Tianjin Medical University Cancer Institute & Hospital, The Fourth Hospital of Hebei Medical University and Tianjin Cancer Hospital Airport Hospital between October 2018 and December 2022 were included in this multicenter, retrospective study. The inclusion criteria were as follows: (1) age ≥ 18, (2) histopathology-proven stage III NSCLC and (3) treatment with neoadjuvant chemoimmunotherapy followed by surgery or CRT. The exclusion criteria included (1) patients with mutant driver genes, such as epidermal growth factor receptor (EGFR) mutations or anaplastic lymphoma kinase (ALK) rearrangements, (2) history of any cancer-specific treatment, (3) tumor progression before immunotherapy and (4) immunotherapy concurrent with radiotherapy (RT) or as part of a clinical trial.

All patients were assessed by a multidisciplinary team (MDT), including thoracic surgeons, radiation oncologists, radiologists, etc., before treatment and after induction therapy, with further consideration of patient treatment preferences to determine the subsequent treatment modality. In general, our MDT team prefers surgery for patients with more localized lesions and single-station lymph node metastases, while CRT for patients with multiple-station or bulky N2. According to the treatment modality after chemoimmunotherapy, the cohort of patients was divided into a surgery group and a CRT group. The patients’ baseline demographic and therapeutic data were extracted from their medical records. Individual NSCLC cases’ histological type and stage were determined according to the WHO criteria [20] and the International Association for the Study of Lung Cancer classification (8th edition) [21], respectively. The age-adjusted Charlson comorbidity index (aCCI) was used to assess the comorbidities between groups [22]. The cutoff point was determined by the median score.

This study conformed to the provisions of the Declaration of Helsinki (as revised in 2013) and was approved by the institutional medical ethics committee (No. bc2023063).

### Drug treatment

The PD-1/PD-L1 inhibitors used including atezolizumab, camrelizumab, durvalumab, nivolumab, pembrolizumab, penpulimab, sintilimab, tislelizumab or toripalimab. These nine kinds of immune checkpoint inhibitors (ICIs) have been approved for the treatment of NSCLC based on the promising outcomes in NSCLC patients [4, 7, 23–29]. Different chemotherapy regimens for patients were adopted depending on the histological type of the tumor, the individual clinical condition of the patient, etc.

### Study outcomes

Clinical outcomes, including PFS and OS, were assessed. PFS was estimated from the start of neoadjuvant treatment to the date of first documented event of disease progression, death without progression or last follow-up. OS was calculated from the initiation of neoadjuvant treatment until death or last follow-up. Patients were followed up every 3 months for the first 2 years after surgery or CRT and every 6 months thereafter. Individual patients’ treatment-related adverse events (TRAEs) were evaluated according to CTCAE version 5.0. In the exploratory analysis, in order to better compare with the PACIFIC trial, we further calculated PFS and OS from the end of CRT in patients who completed CRT without PD.

### Statistical analysis

Patient characteristics were analyzed between treatment groups using the Chi-square test or Fisher’s exact test for categorical variables. The Kaplan‒Meier method was used to estimate PFS and OS, which were then evaluated using the log-rank test. When the univariate analysis yielded a *P* value of < 0.15, the variable was incorporated into the multivariate Cox regression analysis. One-to-one propensity score matching (PSM) with a caliper of 0.02 was performed to further minimize confounding. Propensity scores were calculated based on age, sex, WHO histology, stage, adjuvant ICI, ECOG performance status and aCCI. Subgroup analyses for PFS and OS were performed to assess the consistency of treatment effects in patient subgroups. Subgroup analyses used an unstratified Cox proportional hazards model with treatment as a covariate. *P* value inferior to 0.05 was considered statistically significant. ALL statistical analyses were performed using SPSS version 25 (IBM, Armonk, NY, USA).

## Results

### Baseline characteristics

A total of 239 consecutive eligible patients were included in this study. Among them, 104 (43.5%) received surgery and 135 (56.5%) received CRT. The median age was 63 years (range 27–78). More patients in the surgery group had an ECOG PS score of 0 and fewer comorbidities. Detailed clinical characteristics are shown in Table 1.

**Table 1:** Baseline demographic and clinical characteristics of the patients

Characteristic	Surgery (n = 104)		CRT (n = 135)		
	No	%	No	%	*P*
Age					
< 65	67	64.4	76	56.3	0.204
≥ 65	37	35.6	59	43.7	
Sex					
Male	86	82.7	123	91.1	0.051
Female	18	17.3	12	8.9	
WHO histology					
Squamous	73	70.2	97	71.9	0.079
Non-squamous	30	28.8	30	22.2	
NOS	1	1.0	8	5.9	
T					
1	12	11.5	8	5.9	0.428
2	40	38.5	53	39.5	
3	28	26.9	36	26.7	
4	24	23.1	38	28.1	
N					
0	5	4.8	8	5.9	0.125
1	8	7.7	9	6.7	
2	72	69.2	76	56.3	
3	19	18.3	42	31.1	
Stage					
IIIA	52	50.0	54	40.0	0.095
IIIB	46	44.2	63	46.7	
IIIC	6	5.8	18	13.3	
Adjuvant ICI					
No	56	53.8	86	63.7	0.124
Yes	48	46.2	49	36.3	
ECOG					
0	23	22.1	7	5.2	< 0.001
1	81	77.9	122	90.4	
2	0	0.0	6	4.4	
aCCI					
≤ 2	62	59.6	62	45.9	0.036
> 2	42	40.4	73	54.1	

*CRT* chemoradiotherapy, *NOS* not otherwise specified, *ICI* immune checkpoint inhibitor, *ECOG* Eastern Cooperative Oncology Group, *aCCI* age-adjusted Charlson comorbidity index

### Treatment

All patents were treated with neoadjuvant chemoimmunotherapy and the immune checkpoint inhibitors (ICIs) included atezolizumab (0.4%, n = 1), camrelizumab (9.2%, n = 22), durvalumab (1.3%, n = 3), nivolumab (5.9%, n = 14), pembrolizumab (25.1%, n = 60), penpulimab (0.8%, n = 2), sintilimab (37.2%, n = 89), tislelizumab (18.4%, n = 44) and toripalimab (1.7%, n = 4).

In the surgery group, after receiving a median of 3 cycles of neoadjuvant immunotherapy (range 1–6), all patients received sleeve resection, lobectomy or pneumonectomy combined with routine mediastinal lymphadenectomy, except 2 patients received open thoracotomy. Three patients in the surgery group did not undergo complete resection, while the remaining 101 patients had R0 resection status. One of these three patients subsequently received postoperative radiotherapy, one received subsequent chemoimmunotherapy and one discontinued treatment. Forty-eight (46.2%) patients further received a median of 4 cycles of adjuvant immunotherapy (range 1–24), and 6 (5.8%) patients received postoperative radiotherapy. The type of ICI used for adjuvant immunotherapy was the same as that used preoperatively, with only one patient using the PD-L1 inhibitor and the remaining 47 patients using the PD-1 inhibitor. Among the 48 patients, 14 (29.2%) completed 1 year of adjuvant immunotherapy, and 34 discontinued due to disease progression (22.9%, n = 11), adverse events (10.4%, n = 5), economic burden (18.8%, n = 9) and patient request (18.8%, n = 9).

In the CRT group, after receiving a median of 4 cycles of neoadjuvant immunotherapy (range 1–12), 90 patients received sequential CRT (sCRT), while the remaining 45 patients received cCRT. All patients received a median dose of 60 Gy (range 45–66.0) of radiotherapy, and the majority of chemotherapy regimens were platinum-based doublet chemotherapy (95.6%, n = 129). Forty-nine (36.3%) patients further received adjuvant immunotherapy. Similarly, the type of ICI used for adjuvant immunotherapy after CRT was consistent with that used before CRT, with only two patients using the PD-L1 inhibitor and the remaining 47 patients using the PD-1 inhibitor. Among them, 15 (30.6%) completed 1 year of adjuvant immunotherapy, 34 discontinued due to disease progression (26.5%, n = 13), adverse events (8.2%, n = 4), economic burden (16.3%, n = 8), patient request (12.2%, n = 6) and 3 patients are still under treatment (6.1%).

### Efficacy and safety

In the whole population, the median follow-up from the initiation of neoadjuvant treatment was 26.0 months (range 3.7–60.9 months). Median PFS was 26.1 months, and median OS was not reached (NR). As data cutoff, 115 patients had progressive disease (PD), with 45 (43.3%) in the surgery group and 70 (51.9%) in the CRT group. Of these 115 patients who progressed, one in the surgery group and four in the CRT group progressed during neoadjuvant chemoimmunotherapy. The patient in the surgery group underwent open thoracotomy followed by chemoimmunotherapy, and three of the four patients in the CRT group received sCRT instead of planned surgery. Fifty patients had died at the time of analysis, including 17 (16.3%) in the surgery group and 33 (24.4%) in the CRT group. All patients died of lung cancer, except one patient in the surgery group who died of postoperative pulmonary embolism and four patients in the CRT group who died of other causes. Three of these four patients in the CRT group died of COVID-19 infection, heart disease and accident, respectively, and none of them had tumor progression, while the other patient died of COVID-19 infection after locoregional progression.

The median PFS, 1- and 2-year PFS rates were NR, 75.0% and 61.4% in the surgery group, and 23.2 months, 76.7% and 45.1% in the CRT group. While the median OS, 1- and 2-year OS rates were NR, 96.2% and 89.9% in the surgery group, and 46.0 months, 92.5% and 78.8% in the CRT group. In the exploratory analysis, median PFS and OS from the end of CRT for patients who completed CRT without PD were 17.7 and 40.6 months, respectively. One- and 2-year PFS rates were 64.3% and 40.7%, respectively, while 1- and 2-year OS rates were 88.0% and 78.2%, respectively.

We further compared the incidence of common TRAEs between the two groups. The incidence of TRAEs was similar in both groups, except that the incidence of grade 3/4 hematological toxicity was significantly higher in the CRT group (Table 2). Three patients (2.9%) in the surgery group developed esophagitis following postoperative radiotherapy. Of note, one patient developed a postoperative pulmonary embolism and 38.5% (40/104) of patients in the surgery group developed postoperative pneumonia, which resolved after antibiotic therapy.

**Table 2:** TRAEs between the surgery and CRT groups

TRAE	Surgery		CRT		
	No	%	No	%	*P*
Pneumonitis	67	64.4	89	65.9	0.809
G3/4 pneumonitis	6	5.8	16	11.9	0.107
Postoperative pneumonia	40	38.5	0	0.0	
Esophagitis	3	2.9	30	22.2	
G3/4 esophagitis	0	0.0	0	0.0	
Hematologic toxicity	63	60.6	89	65.9	0.394
G3/4 hematologic toxicity	8	7.7	24	17.8	0.023
Dermatitis	4	3.8	5	3.7	1.000
G3/4 dermatitis	1	1.0	1	0.7	1.000

*TRAEs* treatment-related adverse events, *CRT* chemoradiotherapy; G3/4, grade 3/4

### Radical surgery vs. definitive cCRT

We further investigated the prognostic impact of different radical treatments after neoadjuvant chemoimmunotherapy. After excluding patients with disease progression during neoadjuvant chemoimmunotherapy and restricting to those who underwent radical surgery/definitive cCRT, a total of 143 patients were included, of whom 101 underwent radical surgery (rSurgery group) and 42 underwent definitive cCRT (dCCRT group). Baseline characteristics of both patient groups are detailed in Table 3.

**Table 3:** Baseline characteristics between the rSurgery and dCCRT groups

Characteristics	Before PSM		*P*	After PSM		*P*
	rSurgery *(*n = 101)	dCCRT (n = 42)		rSurgery *(*n = 40)	dCCRT (n = 40)	
	No (%)	No (%)		No (%)	No (%)	
Age						
< 65	66(65.3)	29(69.0)	0.669	27(67.5)	27(67.5)	1.000
≥ 65	35(34.7)	13(31.0)		13(32.5)	13(32.5)	
Sex						
Male	84(83.2)	39(92.9)	0.128	38(95.0)	37(92.5)	1.000
Female	17(16.8)	3(7.1)		2(5.0)	3(7.5)	
WHO histology						
Squamous	71(70.3)	29(69.0)	0.013	26(65.0)	28(70.0)	0.066
Non-squamous	29(28.7)	8(19.0)		14(35.0)	8(20.0)	
NOS	1(1.0)	5(11.9)		0(0.0)	4(10.0)	
Stage						
IIIA	51(50.5)	20(47.6)	0.076	18(45.0)	20(50.0)	0.133
IIIB	45(44.6)	15(35.7)		20(50.0)	13(32.5)	
IIIC	5(5.0)	7(16.7)		2(5.0)	7(17.5)	
Adjuvant ICI						
No	54(53.5)	26(61.9)	0.355	24(60.0)	24(60.0)	1.000
Yes	47(46.5)	16(38.1)		16(40.0)	16(40.0)	
ECOG						
0	23(22.8)	1(2.4)	0.001	1(2.5)	1(2.5)	1.000
1	78(77.2)	40(95.2)		39(97.5)	39(97.5)	
2	0(0.0)	1(2.4)		0(0.0)	0(0.0)	
aCCI						
≤ 2	60(59.4)	23(54.8)	0.608	21(52.5)	22(55.0)	0.823
> 2	41(40.6)	19(45.2)		19(47.5)	18(45.0)	

*rSurgery* radical surgery, *dCCRT* definitive concurrent chemoradiotherapy, *PSM* propensity score matching, *NOS* not otherwise specified, *ICI* immune checkpoint inhibitor, *ECOG* Eastern Cooperative Oncology Group, *aCCI* age-adjusted Charlson comorbidity index

**Fig. 1:**
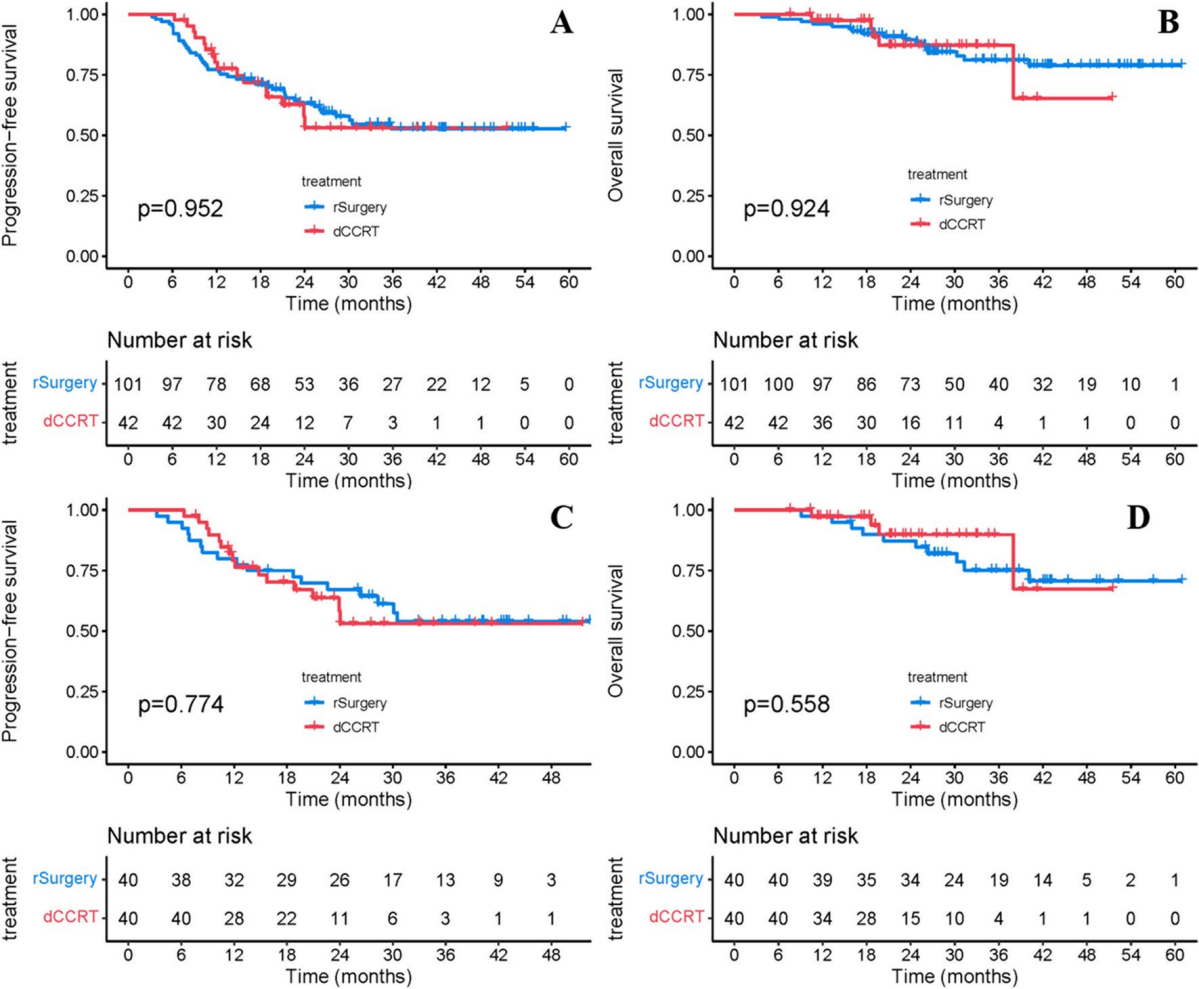
PFS and OS between the rSurgery and dCCRT groups before and after PSM. **a** PFS from the initiation of neoadjuvant treatment before PSM. **b** OS from the initiation of neoadjuvant treatment before PSM. **c** PFS from the initiation of neoadjuvant treatment after PSM. **d** OS from the initiation of neoadjuvant treatment after PSM. *PFS* progression-free survival, *OS* overall survival, *dCCRT* definitive concurrent chemoradiotherapy; rSurgery, radical surgery, *PSM* propensity score matching

**Fig. 2:**
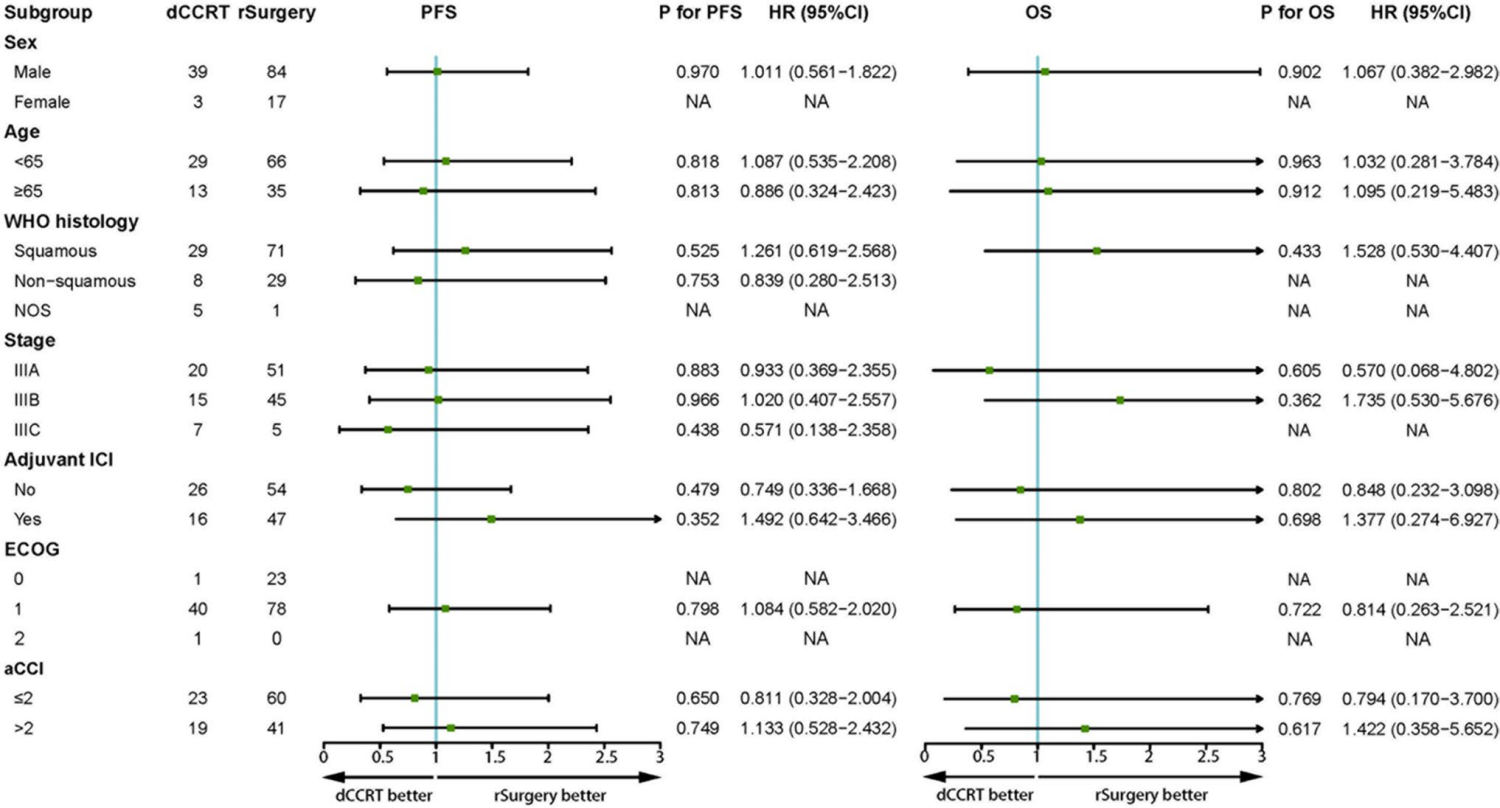
Subgroup analyses of prognostic factors for PFS and OS in patients receiving radical treatment. *PFS* progression-free survival; OS, overall survival, *dCCRT* definitive concurrent chemoradiotherapy; rSurgery, radical surgery, *NOS* not otherwise specified, *ICI* immune checkpoint inhibitor, *ECOG* Eastern Cooperative Oncology Group, *aCCI* age-adjusted Charlson comorbidity index

Median PFS was NR in either group, with a 1-year PFS rate of 77.2% in the rSurgery group vs. 80.2% in the dCCRT group and a 2-year PFS rate of 63.2% vs. 53.0% (*P* = 0.952, Fig. 1A). Median OS were all NR, with a 1-year OS rate of 96.0% vs. 97.5% and a 2-year OS rate of 89.6% vs. 87.1% (*P* = 0.924, Fig. 1B). Univariate and multivariate analyses further confirmed that the choice of radical treatment after neoadjuvant chemoimmunotherapy was not an independent factor affecting PFS (HR = 0.872, *P* = 0.663, Table S1 in the supplementary material) and OS (HR = 0.917, *P* = 0.882, Table S2 in the supplementary material). Further subgroup analyses showed no advantage in PFS or OS favoring either radical surgery or definitive cCRT after neoadjuvant chemoimmunotherapy across patient demographic characteristics and baseline clinicopathological features (Fig. 2).

After a further 1:1 PSM, baseline characteristics between the two treatment groups were basically balanced (Table 3). Still, there was no significant difference in PFS and OS between the rSurgery group and the dCCRT group. One- and 2-year PFS rates were 80.0% vs. 79.2% and 67.2% vs. 53.1%, respectively (*P* = 0.774, Fig. 1C). While 1- and 2-year OS rates were 97.5% vs. 97.4% and 87.3% vs. 89.9%, respectively (*P* = 0.558, Fig. 1D).

Regarding the pattern of relapse, there were three patients in the rSurgery group and one patient in the dCCRT group had unclear patterns of relapse. After removing patients with unknown patterns of relapse, there was no significant difference between the two treatment groups before PSM. While in the matched population, patients in the dCCRT group showed a numerically lower incidence of distant metastases compared to those in the rSurgery group (42.9% vs. 70.6%, *P* = 0.119, Table 4).

**Table 4:** Failure patterns between the rSurgery and dCCRT groups

Failure pattern	rSurgery		dCCRT		
	No	%	No	%	*P*
Before PSM					
Locoregional	32	82.1	11	78.6	1.000
Distant	20	51.3	6	42.9	0.589
1-year locoregional	18	18.6	6	15.8	0.705
1-year distant	9	9.3	4	10.5	1.000
After PSM					
Locoregional	13	76.5	11	78.6	1.000
Distant	12	70.6	6	42.9	0.119
1-year locoregional	7	17.9	6	16.7	0.883
1-year distant	5	12.8	4	11.1	1.000

*rSurgery* radical surgery, *dCCRT* definitive concurrent chemoradiotherapy, *PSM* propensity score matching, *Locoregional* the incidence of locoregional recurrence; *Distant* the incidence of distant metastases, *1-year locoregional* 1-year locoregional recurrence rate, *1-year distant* 1-year distant metastasis rate

In terms of the incidence of TRAEs, the incidence of grade 3/4 hematological toxicity remained higher in the dCCRT group compared to the rSurgery group in the matched population (30.0% vs. 10.0%, *P* = 0.025, Table 5), while the incidence of other TRAEs was not significantly different between the two groups.

**Table 5:** TRAEs between the rSurgery and dCCRT groups after PSM

TRAE	rSurgery		dCCRT		*P*
	No	%	No	%	
Pneumonitis	25	62.5	25	62.5	1.000
G3/4 pneumonitis	2	5.0	2	5.0	1.000
Postoperative pneumonia	16	40.0	0	0.0	
Esophagitis	0	0.0	7	17.5	
G3/4 esophagitis	0	0.0	0	0.0	
Hematologic toxicity	21	52.5	29	72.5	0.065
G3/4 hematologic toxicity	4	10.0	12	30.0	0.025
Dermatitis	1	2.5	1	2.5	1.000
G3/4 dermatitis	0	0.0	0	0.0	NA

*TRAEs* treatment-related adverse events, *rSurgery* radical surgery, *dCCRT* definitive concurrent chemoradiotherapy; G3/4, grade 3/4

## Discussion

The findings of this real-world study demonstrate comparable outcomes between definitive concurrent chemoradiotherapy and radical surgery following neoadjuvant chemoimmunotherapy in stage III NSCLC. These results are very interesting and should be considered hypothesis generating for future evaluation of these treatment modalities as neoadjuvant chemoimmunotherapy is increasingly used in clinical practice for stage III NSCLC, but evidence on the optimal treatment after neoadjuvant chemoimmunotherapy remains limited. To the best of our knowledge, this is the first study to directly compare the efficacy and safety of surgery with chemoradiotherapy after neoadjuvant chemoimmunotherapy through real-world data.

Debate over optimal treatment for stage III NSCLC does not appear to be diminishing with the advent of immunotherapy. Whether patients are operable or inoperable, immunotherapy in combination with surgery or CRT can significantly improve survival and has become the dominant treatment modality for stage III NSCLC [3, 7, 9, 30]. Although several decision-making surveys in stage III NSCLC have shown that surgery is the preferred option for patients with non-bulky mediastinal lymph node involvement or single-station N2, and CRT is the preferred option for patients with more extensive lymph node involvement [31–33], the impressive efficacy of neoadjuvant immunotherapy is gradually obscuring the already ill-defined concept of resectability and has led to its gradual application in potentially resectable and even unresectable NSCLC. Notably, a recent study highlighted the importance of dLNs in antitumor immune responses in humans [15], raising the question of whether there is a negative effect on antitumor immunity of regional lymphadenectomy in radical surgery or whether there is an option that can be an alternative to radical surgery involving regional lymphadenectomy in stage III NSCLC. In this study, the 2-year PFS rate of approximately 61% and the 2-year OS rate of approximately 89% of patients who underwent surgery after neoadjuvant chemoimmunotherapy in this study were similar to previous clinical trials [7, 9, 34]. And patients in the CRT group also showed comparable survival to those in the PACIFIC trial. Median PFS and 2-year PFS rate calculated from the end of CRT (17.7 months and 40.7%, respectively) were similar to those reported in the PACIFIC trial (16.9 months and 45.0%, respectively). Median OS and 2-year OS rate (40.6 months and 78.2%, respectively) were also noninferior to that from the PACIFIC trial (47.5 months and 66.3%, respectively) [4]. These survival data demonstrate comparable real-world efficacy of these two treatment modalities to the results of the clinical trials that established the current dominant treatment paradigm for stage III NSCLC.

Previous studies have demonstrated the superiority of cCRT to sCRT in patients with stage III NSCLC in terms of OS and reduced risk of locoregional progression, even after neoadjuvant chemoimmunotherapy [35–37]. However, to date, no phase III randomized trial has evaluated the application of immunotherapy in multimodal treatment regimens for patients with stage III NSCLC stratified by definitive treatment with surgery or CRT. Only a phase II trial conducted by Wu et al. found that surgery after neoadjuvant chemoimmunotherapy appeared to be superior to definitive cCRT (1-year EFS 74.4% vs. 55.9%), but they focused on a highly selected population stratified by PD-L1 expression and followed by an MDT to determine subsequent treatment [12]. Considering that patients receiving definitive cCRT in this study accounted for only 1/3 of patients receiving CRT, the application of definitive cCRT and radical surgery after neoadjuvant chemoimmunotherapy was further investigated. In accordance with the results in the preimmunotherapy era [18, 19], our results indicate that definitive cCRT was noninferior to radical surgery after neoadjuvant chemoimmunotherapy, even across all subgroups. The observed 1- and 2-year PFS rates in patients receiving definitive cCRT were 79.2% and 53.1%, respectively, while the 1- and 2-year OS rates were 97.4% and 89.9%, respectively, noninferior to the results of the AFT-16 (66% for 1-year PFS rate) and KEYNOTE-799 (approximately 60% for 2-year PFS rate and approximately 70% for 2-year OS rate) trials evaluating the safety and efficacy of immunotherapy before radiotherapy [23, 38].

Interestingly, patients receiving definitive cCRT demonstrated a similar incidence of locoregional recurrence but a numerically lower incidence of distant metastases compared to those receiving radical surgery, which is different from previous studies in the preimmunotherapy era [39, 40]. Previous studies showed that patients who received radiotherapy, even with stereotactic ablative radiotherapy (SABR), still had a higher incidence of locoregional recurrence, although the incidence of distant metastases appeared to have decreased compared to surgery [18, 39]. Of note, in operable stage I NSCLC patients who received SABR, the 3-year regional recurrence-free survival was 90% and the 3-year metastasis-free survival was 97% in Chang’s pooled study, yet the 2-year regional recurrence-free survival decreased to 53%, and the 2-year metastasis-free survival decreased to 76% in patients who received surgery after SABR [39, 41]. Combined with recent studies highlighting the importance of dLNs, we therefore speculate that the similar incidence of locoregional recurrence and relatively lower incidence of distant failure in our dCCRT group compared to the rSurgery group may be correlated with the addition of neoadjuvant chemoimmunotherapy and that definitive cCRT may preserve regional lymph nodes better than radical surgery and thus protect antitumor immunity. The 12-month incidence of locoregional recurrence and distant metastases in this study was 16.7% and 11.1%, respectively, which appears to be better than previous studies on the failure patterns of the PACIFIC regimen (approximately 20% for 12-month locoregional recurrence and approximately 30% for 12-month distant metastasis) [42–44]. Taken together, definitive cCRT may achieve noninferior outcomes to radical surgery after neoadjuvant chemoimmunotherapy with a numerically decreased incidence of distant failure.

Although the two different local treatment modalities resulted in a slightly different spectrum of TRAEs in the two treatment groups in this study, the common TRAEs were still relatively similar between the two treatment groups. There was no significant difference in the incidence of common TRAEs in either the whole population or the matched population, except for grade 3/4 hematological toxicity, which was higher but still acceptable in patients receiving CRT, suggesting that the safety of the two treatment modalities is relatively comparable. Regarding the incidence of pneumonia, which is of great concern, we note that although there was no significant difference in the incidence of pneumonia between the two groups, more than one-third of patients who underwent surgery developed postoperative pneumonia and were treated with antibiotics, and that the majority of patients received antibiotics in the perioperative period. However, there is increasing evidence that the application of antibiotics may reduce the efficacy of immunotherapy by disrupting the balance of gut microbiome [45–47]. It is unclear whether the perioperative application of antibiotics contributed to the similar outcomes of the two radical treatment modalities in this study, and large prospective clinical trials are needed to validate these findings in the future.

It is worth noting that although definitive cCRT following neoadjuvant chemoimmunotherapy appears to be noninferior to radical surgery in the present study, it must be acknowledged that the poor proportion of definitive cCRT also reflects to some extent the real-world treatment patterns of stage III NSCLC. A web-based survey of US oncologists found that an average of about 69% of patients with new unresectable stage III NSCLC receive radiotherapy, with only 48% receiving cCRT [48]. Moreover, in a prospective multicenter study investigating real-world treatment patterns for stage III NSCLC in China, only 41.3% (142/344) of patients with unresectable disease received CRT, and 62.7% (89/142) of these patients received cCRT. In contrast, all patients with resectable disease received surgery [49]. More importantly, patients who are inoperable or refuse surgery may not be able to tolerate definitive cCRT. Nevertheless, the key highlight of this study is the combination of immunotherapy, surgery and radiotherapy, which may break the barrier of the seemingly opposite sequence of immunotherapy application for resectable versus unresectable stage III NSCLC. Although several studies have shown the efficacy and safety of upfront immunotherapy before radiotherapy, the current standard of care for unresectable stage III NSCLC remains the PACIFIC regimen due to the absence of direct comparison with the PACIFIC regimen [23, 38, 50, 51]. However, recently published population-based study demonstrated better survival after surgery following neoadjuvant chemoimmunotherapy than cCRT followed by immunotherapy in patients with stage III-N2 NSCLC [52]. The comparable outcomes of the two radical treatment modalities after neoadjuvant chemoimmunotherapy in the present study suggest that patients with stage III NSCLC, whether resectable or unresectable, may benefit equally from several cycles of neoadjuvant chemoimmunotherapy followed by either radical treatment. The addition of neoadjuvant chemoimmunotherapy could prolong survival in patients with resectable NSCLC, while in patients with unresectable NSCLC, it could downsize initial tumors and contribute to following definitive CRT [7–9, 50], and those who receive definitive cCRT may achieve noninferior outcomes to those who receive radical surgery. Moreover, combining with adjuvant immunotherapy may further improve the outcome.

It should be pointed out that the present study has several limitations. First, this is a real-world retrospective study, and the inherent drawbacks of retrospective studies made some selection bias inevitable, while the lack of original imaging data in a proportion of patients made it difficult to further explore the prognostic impact of the two treatment modalities based on different N2 status, such as bulky or multi-station N2. In addition, the moderate sample size of patients receiving definitive cCRT limited the ability to draw a definitive conclusion, but our study provided the preliminary evidence for future large-scale and prospective trials. Furthermore, data regarding PD-L1 expression were sparse because it is not routinely tested in stage III NSCLC at our three centers. Meanwhile, this study did not further differentiate between the efficacy of various ICIs. Nevertheless, this multicenter study reflects to some extent the use and benefits of immunotherapy in stage III NSCLC in the real-world practice. Future studies should use the same ICI agent and stratify by PD-L1 expression to minimize confounding. Despite these limitations, this is the first study to investigate different treatment modalities after neoadjuvant chemoimmunotherapy in stage III NSCLC through real-world data. We believe this study may provide a new direction for research or an option for stage III NSCLC patients who refuse or cannot receive surgery after neoadjuvant chemoimmunotherapy.

## Conclusions

Following neoadjuvant chemoimmunotherapy, definitive concurrent chemoradiotherapy may achieve noninferior outcomes to radical surgery. Prospective randomized trials are warranted to further validate these findings.

### Supplementary Information

Below is the link to the electronic supplementary material.

Supplementary file 1 (DOCX 393 KB)

## Data Availability

Data will be made available on request.
