# Supplementary material for: Chemoradiotherapy versus surgery after neoadjuvant chemoimmunotherapy in patients with stage III NSCLC: a real-world multicenter retrospective study
Source: Cancer Immunol Immunother. 2024 May 7;73(7):120. doi: 10.1007/s00262-024-03696-4 (PMC11076427; doi:10.1007/s00262-024-03696-4)
Supplement: Supplementary file 1 — (DOCX 393 KB) [file 262_2024_3696_MOESM1_ESM.docx]

**Table S1.** Univariate and multivariate analyses for PFS in patients receiving radical treatment

| Factor | Univariate | |  | Multivariate | |  |
| --- | --- | --- | --- | --- | --- | --- |
|  | HR | 95% CI | *P* | HR | 95% CI | *P* |
| Age |  |  |  |  |  |  |
| <65 | 1.000 (reference) | | |  | | |
| ≥65 | 1.121 | 0.657, 1.910 | 0.676 |  |  |  |
| Sex |  |  |  |  |  |  |
| Male | 1.000 (reference) | | | 1.000 (reference) | | |
| Female | 0.440 | 0.176, 1.101 | 0.079 | 0.411 | 0.163, 1.032 | 0.058 |
| WHO histology |  |  |  |  |  |  |
| Squamous | 1.000 (reference) | | | 1.000 (reference) | | |
| Non-squamous | 1.539 | 0.895, 2.647 | 0.119 | 1.343 | 0.777, 2.320 | 0.291 |
| NOS | 0.382 | 0.052, 2.782 | 0.342 | 0.312 | 0.041, 2.374 | 0.261 |
| Stage |  |  |  |  |  |  |
| IIIA | 1.000 (reference) | | | 1.000 (reference) | | |
| IIIB | 1.260 | 0.727, 2.182 | 0.410 | 1.359 | 0.781, 2.365 | 0.277 |
| IIIC | 3.490 | 1.551, 7.853 | 0.003 | 4.116 | 1.748, 9.693 | 0.001 |
| Adjuvant ICI |  |  |  |  |  |  |
| No | 1.000 (reference) | | |  | | |
| Yes | 0.999 | 0.597, 1.670 | 0.996 |  |  |  |
| ECOG |  |  |  |  |  |  |
| 0 | 1.000 (reference) | | |  | | |
| 1 | 0.714 | 0.378, 1.348 | 0.298 |  |  |  |
| 2 | 2.167 | 0.280, 16.790 | 0.459 |  |  |  |
| aCCI |  |  |  |  |  |  |
| ≤2 | 1.000 (reference) | | | 1.000 (reference) | | |
| >2 | 1.548 | 0.929, 2.579 | 0.094 | 1.604 | 0.958, 2.684 | 0.072 |
| Treatment |  |  |  |  |  |  |
| rSurgery | 1.000 (reference) | | | 1.000 (reference) | | |
| dCCRT | 1.018 | 0.571, 1.814 | 0.952 | 0.872 | 0.470, 1.618 | 0.663 |

*Abbreviations:* PFS, progression-free survival; rSurgery, radical surgery; dCCRT, definitive concurrent chemoradiotherapy; NOS, not otherwise specified; ICI, immune checkpoint inhibitor; ECOG, Eastern Cooperative Oncology Group; aCCI, age-adjusted Charlson comorbidity index.

**Table S2.** Univariate and multivariate analyses for OS in patients receiving radical treatment

| Factor | Univariate | |  | Multivariate | |  |
| --- | --- | --- | --- | --- | --- | --- |
|  | HR | 95% CI | *P* | HR | 95% CI | *P* |
| Age |  |  |  |  |  |  |
| <65 | 1.000 (reference) | | |  | | |
| ≥65 | 1.357 | 0.580, 3.175 | 0.482 |  |  |  |
| Sex |  |  |  |  |  |  |
| Male | 1.000 (reference) | | |  | | |
| Female | 0.261 | 0.035, 1.944 | 0.190 |  |  |  |
| WHO histology |  |  |  |  |  |  |
| Squamous | 1.000 (reference) | | |  | | |
| Non-squamous | 0.537 | 0.181, 1.588 | 0.261 |  |  |  |
| NOS | 0.000 | 0.000 | 0.979 |  |  |  |
| Stage |  |  |  |  |  |  |
| IIIA | 1.000 (reference) | | | 1.000 (reference) | | |
| IIIB | 2.050 | 0.848, 4.952 | 0.111 | 1.927 | 0.779, 4.766 | 0.156 |
| IIIC | 0.896 | 0.112, 7.177 | 0.918 | 0.931 | 0.113, 7.659 | 0.947 |
| Adjuvant ICI |  |  |  |  |  |  |
| No | 1.000 (reference) | | |  | | |
| Yes | 0.729 | 0.306, 1.739 | 0.477 |  |  |  |
| ECOG |  |  |  |  |  |  |
| 0 | 1.000 (reference) | | | 1.000 (reference) | | |
| 1 | 0.980 | 0.329, 2.920 | 0.970 | 0.928 | 0.298, 2.889 | 0.897 |
| 2 | 12.607 | 1.334,119.177 | 0.027 | 9.255 | 0.727,117.751 | 0.086 |
| aCCI |  |  |  |  |  |  |
| ≤2 | 1.000 (reference) | | |  | | |
| >2 | 1.277 | 0.553, 2.946 | 0.567 |  |  |  |
| Treatment |  |  |  |  |  |  |
| rSurgery | 1.000 (reference) | | | 1.000 (reference) | | |
| dCCRT | 1.050 | 0.382, 2.885 | 0.924 | 0.917 | 0.295, 2.856 | 0.882 |

*Abbreviations:* OS, overall survival; rSurgery, radical surgery; dCCRT, definitive concurrent chemoradiotherapy; NOS, not otherwise specified; ICI, immune checkpoint inhibitor; ECOG, Eastern Cooperative Oncology Group; aCCI, age-adjusted Charlson comorbidity index.
